# Climate crisis and ecological emergency: Why they concern (neuro)scientists, and what we can do

**DOI:** 10.1177/23982128221075430

**Published:** 2022-02-28

**Authors:** Charlotte L. Rae, Martin Farley, Kate J. Jeffery, Anne E. Urai

**Affiliations:** 1 School of Psychology, University of Sussex, Falmer, UK; 2 Sustainable UCL, University College London, London, UK; 3 Research Management & Innovation Directorate, King’s College London, London, UK; 4 Division of Psychology and Language Sciences, University College London, London, UK; 5 Cognitive Psychology Unit, Leiden University, Leiden, The Netherlands

**Keywords:** Climate crisis, ecology, global warming, sustainability, scientific practice, laboratory procedures, conferences, advocacy

## Abstract

Our planet is experiencing severe and accelerating climate and ecological breakdown caused by human activity. As professional scientists, we are better placed than most to understand the data that evidence this fact. However, like most other people, we ignore this inconvenient truth and lead our daily lives, at home and at work, as if these facts weren’t true. In particular, we overlook that our own neuroscientific research practices, from our laboratory experiments to our often global travel, help drive climate change and ecosystem damage. We also hold privileged positions of authority in our societies but rarely speak out. Here, we argue that to help society create a survivable future, we neuroscientists can and must play our part. In April 2021, we delivered a symposium at the British Neuroscience Association meeting outlining what we think neuroscientists can and should do to help stop climate breakdown. Building on our talks (Box 1), we here outline what the climate and ecological emergencies mean for us as neuroscientists. We highlight the psychological mechanisms that block us from taking action, and then outline what practical steps we can take to overcome these blocks and work towards sustainability. In particular, we review environmental issues in neuroscience research, scientific computing, and conferences. We also highlight the key advocacy roles we can all play in our institutions and in society more broadly. The need for sustainable change has never been more urgent, and we call on all (neuro)scientists to act with the utmost urgency.

## Introduction

Human activity is destabilising our planetary equilibrium by destroying ecosystems and changing the climate. We are causing climate change by releasing exponentially increasing quantities of greenhouse gases into the atmosphere, thereby warming the planet. Current rates of emissions (‘business as usual’) are projected to push the planet to at least 3°C of heating compared to pre-industrial baseline by the end of the century. Together with direct ecosystem damage from deforestation and pollution, this is having calamitous effects on ecosystems worldwide. Already today, with temperatures at 1.1°C–1.3°C above pre-industrial levels, we are witnessing loss of agricultural land, forests, and fisheries, as well as more frequent extreme weather events such as heat waves, wildfires, floods, droughts, and hurricanes. The near-term (within current lifetimes) projected results of business as usual include crop failures, water shortages, poverty and hunger, mass migration, and conflict (Masson-Delmotte et al., 2018). Rising sea levels threaten many coastal cities, which house around 10% of the world’s population (Hallegatte et al., 2013). Warming oceans are causing widespread loss of marine life, much of which is an important food source. We are undergoing a global collapse in biodiversity, including pollinators that we rely on for food production (Intergovernmental Science-Policy Platform on Biodiversity and Ecosystem Services, 2019). The area of agriculturally productive land is shrinking (Mbow et al., 2019).

**Box 1.** BNA 2021 symposium: ‘Green neuroscience’ (YouTube, slides).

Charlotte Rae – *The environmental impacts of cognitive neuroscience, from liquid helium to big data: what’s our footprint?* Martin Farley – *Sustainable laboratory research: LEAF and green lab efforts* Anne Urai – *Decarbonizing science: action in academic communities and institutions* Kate Jeffery – *Changing minds: how neuroscientists can influence public and political action on the climate and ecological crisis*

Climate breakdown is for practical purposes irreversible: even if we were to cease carbon emissions tomorrow, it will be decades before atmospheric temperatures return to baseline and centuries before the oceans, which are a huge heat sink, cool again, and the ice caps re-form (Masson-Delmotte et al., 2018). Negative emissions technologies do not yet exist that can be deployed at scale on the urgent timeline required (Fuss et al., 2014). Similarly, increasing biological sinks for carbon, such as by mass reforestation, will take decades that we do not have at our disposal (Nolan et al., 2021). Species extinction cannot be reversed. We must therefore focus on immediate prevention, as there is no meaningful cure.

There is also a moral dimension to the climate crisis. Affluent, developed countries are the strongest contributors to global heating (Nielsen et al., 2021), whereas those in the global South, who contributed little to global emissions and are less well-equipped to deal with climate disruptions, most severely suffer the consequences. Furthermore, our generation is the last in a position to prevent irreversible damage that will deprive future generations, starting with our own children, of the habitable, fertile, and biodiverse planet their predecessors have enjoyed.

Collectively, we have known about environmental degradation and global heating for decades but have so far failed to take meaningful action. The psychology behind environmental inaction is complex and includes psychological self-preservation tactics such as denial (‘it isn’t happening’ or ‘it’s happening but isn’t so bad’ or ‘we can fix it easily’), hopelessness (‘the problem is too big’, ‘other people/countries won’t play their part’), or fatalism (‘it’s too late’, ‘humans deserve to go extinct’). Scientists have been notably inert on the subject. For one thing, we have failed to recognise that our scientific activities are contributing to the crisis. Also, being human, we have the same instincts to denial and psychological self-preservation as everyone else. Many scientists harbour a belief that technology will get us out of our predicament: this is because we are trained to think technologically, and to focus on the many successes of science, including those that are helping end the current Covid-19 pandemic. What we tend not to appreciate is the many things science has *failed* to solve: cancer, dementia, infectious disease, addiction, and the nuclear fusion technology we have been promised for so many decades now, to name just a few. Some problems are just too large or complex to solve technologically, and 40 billion tonnes of atmospheric carbon per annum (and rising exponentially) is unfortunately one of them.

The Covid-19 pandemic has only emphasised the fragility of scientific pursuits. Time-delayed systems (such as climate change or pandemic spread) elicit paradoxical human behaviour: we fail to act early due to the large perceived immediate cost, which exacerbates problems and ultimately leads to far higher human and economic costs (Balmford et al., 2020). While many of us may prefer to stay out of politics and focus on our own research, academics are increasingly waking up to the reality that our own work is not isolated from large societal developments (Rillig et al., 2021). We do not exist in a bubble, and neuroscience depends on a stable climate to thrive. Extreme weather events have already caused numerous disruptions to scientific research: from blazing wildfires in California and Australia to heat waves engulfing the Pacific Northwest and severe floods across Europe. One of us experienced weeks of disruption to data collection, when a tropical storm in New York caused prolonged power outages and forced laboratories to close down. These extreme weather events are not a manageable ‘new normal’, but only a harbinger of more extreme climate fluctuations to come. If we do not change course in the next decades, the destabilising effects of environmental catastrophes will severely threaten our ability to pursue neuroscience unhindered. Furthermore, one might reasonably ask what the *point* of neuroscience is, if the brains that we seek to understand are, themselves, under existential threat.

It is clear what needs to be done. By rapidly ceasing our burning of fossil fuels, shifting to renewable energy, cleaning waste streams, and moving towards circular economies, we can avoid the most catastrophic consequences of our current behaviour (Otto et al., 2020). This requires large-scale social and political change, which in turn relies on the individual and collective action of many individuals. Each of us must look beyond our carbon footprint: by focusing only on personal emissions, we risk spending our energy on individual actions that don’t instantiate broader change. Instead, we can consider our ‘climate shadow’, the full impact we have in our interactions with others. Talking about our worries and leading by example in our changes are crucial to change social norms and ultimately influence policy.

**Figure 1.**
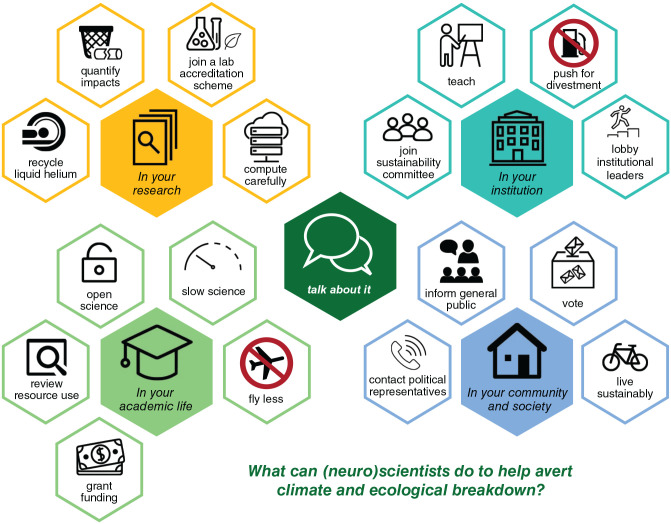
Ways in which (neuro)scientists can act on the climate and ecological emergencies. We distinguish four areas of influence and a non-exhaustive list of specific actions, each of which is discussed in greater detail in the article.

**Figure 2.**
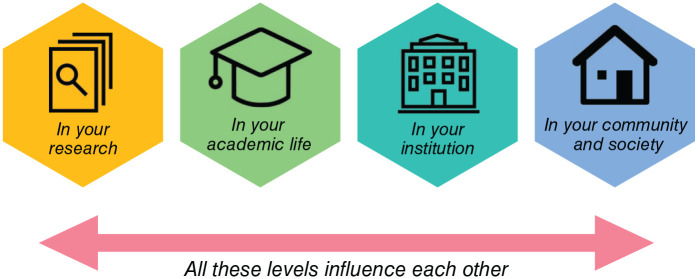
Climate action across spheres of influence. Our day-to-day actions induce social change, creating a mandate for leaders and power-holders to act at higher levels. Societal-level changes and government legislation are ultimately necessary to meaningfully change people’s behaviour at the global scale required.

Here, we discuss how neuroscientists can act (Figure 1; see also Aron et al., 2020 and Zak et al, 2020). We start by detailing local action in our laboratories (from biology to cognitive neuroscience) and day-to-day research activities, and then zoom out to our duties as members of academic institutions and professional communities. We finally discuss scientists’ role in public debate, education, and advocacy. Throughout, we emphasise how all these levels of action co-exist and strengthen each other (Figure 2).

## Environmental footprints of neuroscience research

Neuroscience research has a large environmental footprint, ranging from consumption of experimental resources such as plastics and chemicals in the lab, to the energy associated with infrastructure (buildings and their maintenance), animal housing, equipment manufacture and use, and data storage and analysis. The energy we use and its associated carbon emissions are helping drive the climate crisis, and extraction and disposal of the materials that make up our scientific consumables and equipment play a part in ecosystem damage and biodiversity loss. These environmental costs occur at all stages of the research pipeline. As a community, we need to be much more aware of the ways in which our scientific enterprises harm the planet, and take steps to reduce this harm (see Box 2).

**Box 2.** Steps to tackle environmental costs of neuroscience research.

• **Quantify.** Identify and evaluate the climate and ecological costs of your research. Push suppliers and manufacturers to evaluate and share the environmental impacts of their products via life-cycle assessments. The first step to action is often to understand the scale of impacts. • **Laboratory Practices.** Integrate sustainable lab practices into your research. This includes but is not limited to increasing reuse of consumables, managing equipment in a more sustainable manner, and managing samples and chemical stockpiles. Ensure laboratory practices integrate quality control, to improve conditions for reproducible research. Consider doing this via an accreditation scheme such as LEAF. • **Liquid helium for MRI and MEG scanners.** Helium is a by-product of fossil fuel extraction. Install a helium recycling tank for MEG to capture boil-off and support development of new non-helium methods such as OPM-MEG. • **Computing demands for data analysis and modelling.** Run only analyses and models that you need to, optimise modelling to minimise energy costs, and avoid running jobs at peak times for energy demand. • **Resource usage.** Consider carefully how much data to acquire, analyse, store, and share. Reduce storage of unnecessary files, regularly clean up data, remove intermediary processing stages, and consider how much needs to be stored long term. • **Data sharing.** Where possible, use an open science repository that runs on renewable energy, such as the Open Science Framework. • **Slow science.** Focus on quality over quantity, in line with ‘slow science’ principles (Frith, 2020). • **Engage peers.** Raise awareness of impacts and contribute to community actions to establish best practices where this is currently unknown, such as through the Organization for Human Brain Mapping’s Sustainability and Environment Action Group. The ClimateActionNeuPsych Slack group provides a forum to discuss among colleagues and share best practices in, for example, teaching, conferences, laboratory practice, and institutional policy.

MRI: magnetic resonance imaging, MEG: magnetoencephalography, OPM-MEG: Optically Pumped Magnetometer magnetoencephalography.

### Laboratories: energy consumption, equipment, and standardised mitigation

Wet labs are key facilities for many branches of neuroscience: however, the full environmental impacts of neuroscience laboratories have yet to be fully quantified. This is largely due to the lack of Scope 3 emissions data associated with them, which includes monitoring of indirect emissions such as in supply chains (Box 3). A similar issue arises with consumables such as plastics. Although we still do not know the exact environmental costs of neuroscience laboratories, we do know that they result in significant carbon emissions: this becomes evident when looking at specific institutions. For example, at UCL, where two of us are based, approximately 48% of the whole institution’s emissions derive from science facility operations.

**Box 3.** What are Scope 1, 2, and 3 emissions?

Greenhouse gas emissions are categorised into three groups or ‘Scopes’. • ***Scope 1*** covers *direct emissions* from owned or controlled sources, such as vehicles or institutional power plants. • ***Scope 2*** covers *indirect emissions* from *energy purchased* from a utility provider. • ***Scope 3*** includes *all other indirect emissions* that occur in the supply chain. These range from business travel, waste disposal and purchasing (embodied carbon of materials) to financial investments. These are the most difficult to estimate and tend to make up the majority of an organisation’s emissions.

Despite the lack of comprehensive quantification, there are immediate actions neuroscientists can take to mitigate many known sources of carbon emissions. The largest source of energy consumption within labs typically derives from ventilation (Dockx, 2015). While lab users cannot redesign their ventilation systems, they can take mitigation steps: for example to close the sashes of fume cupboards, which significantly reduces energy consumption (Haugen, 2020). Beyond ventilation, laboratory equipment is often energy-intensive. In a plug-load assessment at the University of Stanford, lab equipment represented 14% of all equipment surveyed, but was responsible for 50% of the energy consumption (Hafer, 2017). Lab users can simply turn off equipment more often, but further reductions will rely on improved equipment operation while balancing research needs. For example, users may consider changing ultra-low temperature storage conditions from −80°C to −70°C, which can save 28% in energy consumption. In doing so, they should consider a variety of factors, such as freezer door-opening frequency, sample location within the units, and sample type.

Laboratory equipment has impacts beyond its energy consumption, albeit, again, Scope 3 data on the embodied carbon associated with manufacturing is often lacking. However, life-cycle assessments (LCAs) on comparable pieces of equipment, such as in refrigeration (Cascini et al., 2013), indicate the significant impacts manufacturing can have on the environment. In this respect, the purchasing, upkeep and maintenance, usage, and disposal of equipment are important factors in the overall sustainability of scientific operations. During purchase, users should seek equipment with the lowest LCA possible and avoid unnecessary replacement unless both the LCA and energy efficiency have been considered. Equipment should be maintained and repaired where feasible, to improve longevity and reduce the need for new purchases. Sometimes equipment is no longer required even though it is still functional: in such cases users should donate or re-sell equipment. Institutions should also provide easy access to repair services, as at UCL where repair clinics have been hosted across the campus. Here, repair teams were sent to various institutes, and lab users could request services on-site at no extra cost (unless further parts were required). Disposal of equipment should only be considered when repair, resale, or options for donation have been exhausted. The responsible use of equipment highlights how meaningful action can only arise from coordinated efforts across levels: local (scientists’ purchasing decisions), institutional (university policies and repair support), and governmental (mandatory standards for equipment lifetimes and energy efficiency) (Figure 2).

Today’s laboratory neuroscience research techniques rely on access to single-use consumables, many of which are plastic. Because plastic is made from oil, these consumables require fossil fuels for manufacture. At the end of their short life, many such lab items are incinerated, or placed in landfill (which can generate even greater emissions), because we have no other way of disposing of them (Rizan et al., 2021). While quantification efforts are underway, wet labs are just starting to assess how to reduce and recycle the consumption of single-use plastics (Alves et al., 2020). Where possible, reusable consumables should be prioritised over single-use disposable items, although considerations for contamination, safety, and throughput must be balanced with environmental impacts. This has resulted in institutions developing their own guidance for wet lab users: examples include UCL, Oxford, and Edinburgh.

Wet lab neuroscientists must consider a multitude of factors to reduce the environmental impact of laboratory operations. Making a lab more ‘green’ can be a complicated endeavour, requiring researchers to independently research what is feasible and review existing case studies. Having a list of predetermined steps can be a powerful mechanism for ensuring staff take action in complex settings, as has been evidenced with safety checklists in clinical surgery (Papadakis et al., 2019). For this reason, Sustainable UCL has developed the LEAF programme. LEAF is a standardised list of actions that any staff or student can implement within a laboratory setting, to mitigate the environmental impact of operations. Criteria include issues listed above (ventilation, equipment, and plastic consumables), but also actions around waste management, procurement, teaching, people, water, and research quality. LEAF is the first programme to establish a connection between research quality and environmental sustainability, in recognition that high quality, reproducible, science is less wasteful. As a result, LEAF is supported by the UK Reproducibility Network. LEAF is currently in use within 53 institutions since launching in February 2021. In recognition of the financial savings and carbon emission reductions possible by implementing LEAF, the tool contains calculators which allow users to quantify such impacts. Many other institutions have their own recognised programmes to improve the sustainability of laboratory operations: examples include University of Colorado, Boulder, the University of Georgia, Emory, Harvard in the United States and the Universities of Bristol and Edinburgh in the United Kingdom. Other initiatives and networks include the UK’s LEAN, the Max Planck Sustainability Network in Germany, Green Your Lab, My Green Lab, I2SL and national networks like Green Lab NL, Green Labs Austria, and Sustainable Labs Canada. With the breadth of tasks at hand for neuroscience researchers, and the complexity of reducing environmental impacts of laboratory operations, such initiatives will become an increasingly important resource in standardising good practice.

In summary, we encourage neuroscientists using laboratories to close the fume hood, consider energy usage and maintenance of equipment, switch to reusable items over single-use consumables where possible, and consider the entire life-cycle costs of equipment from production to disposal. More broadly, it is important to adopt an institutional sustainable labs policy, enrol on a certification programme such as LEAF, and share good practice with fellow scientists around the world (Zak et al., 2020).

### Cognitive neuroscience: hardware and computing

It is not just wet labs that are resource-hungry and polluting; cognitive neuroscience also has a significant environmental impact. Cognitive neuroscience techniques such as magnetic resonance imaging (MRI), magnetoencephalography (MEG), electroencephalography (EEG), positron emission tomography (PET), and transcranial magnetic stimulation (TMS) require specialist equipment , and that has an environmental footprint, both in manufacture and usage. MRI and MEG scanners also require liquid helium to cool the superconducting elements. Helium is a naturally occurring substance in the geological environment, which exists almost entirely in reserves of natural gas, buried in the Earth. This means the only way we obtain helium for our scanners is as a by-product of fossil fuel extraction. If we are to decrease fossil fuel usage by 60% by 2030, and eliminate it by 2050, how will we cool our scanners without the helium buried in natural gas deposits? Many modern MRI scanners, such as the Siemens Prisma, have zero helium boil-off technology that reduces the frequency of topping up helium to once every 10 years. For MEG scanners, which use helium at a faster rate than MRI, labs can install a helium recycling tank to capture boil-off (Takeda et al., 2011). This reduces operating costs and protects laboratories against the wildly fluctuating market prices of mined helium. In the longer term, non-helium brain scanning techniques such as Optically-Pumped Magnetometer MEG (Boto et al., 2018) may eliminate our need for helium completely.

Scientific computing is another surprisingly resource-intensive enterprise. Neuroscientists require ever-increasing compute resources to analyse ever-growing datasets (Glasser et al., 2016; Littlejohns et al., 2020; The International Brain Laboratory et al., 2019), and this brings with it increased energy demands and carbon costs. Data centres and IT equipment are environmentally costly to build and energy-hungry to run (in part due to the requirement for constant air conditioning, even when data are not being analysed). Indeed, in 2017, data centres produced 2% of global carbon emissions, and their absolute carbon footprint continues to grow. In computational neuroscience and machine learning, computing demands can be very substantial (Anthony et al., 2020). Computer scientists are increasingly aware of this issue (Rolnick et al., 2019), and tools such as CodeCarbon (Goyal-Kamal et al., 2021) make it easy to quantify the carbon footprint of a piece of software. Novel, energy-efficient computing hardware (Marković et al., 2020) and software (Schwartz et al., 2020) are under active development in computer science and engineering. However, until these are widely implemented, we should consider carefully how much data to acquire, analyse, store, and share: the more we do, the bigger our footprint. Storage via hard media (such as tapes for human brain imaging) might reduce this energy requirement, but may incur other environmental costs in the manufacture and eventual disposal of the hard media. Ask if your data centre considers the time of day that analyses are run: even in countries with high renewable energy fractions, fossil fuels often supplement renewable generation at peak times. Practising good data management can lower a project’s energy use, while improving scientific quality and reproducibility: for example remove unnecessary intermediary files, use version control to avoid re-running analyses, and test code locally before deploying it on large datasets.

The environmental cost of computing also applies to data sharing, as open science repositories run on servers in data centres. Ultimately, given the environmental costs of acquiring data, it may be that reusing open datasets is the more sustainable approach, and indeed open science practices often save time and resources in general. Some repositories, like the Open Science Framework (OSF) which uses Google Cloud, are run using 100% renewable energy. FigShare, as well as other popular repositories for human brain imaging research, such as OpenNeuro (for sharing raw data) and NeuroVault (for sharing statistical results), use Amazon Web Services (AWS), which in 2020 used only 50% renewables. While AWS is ‘committed to achieving 100% renewable energy usage by 2025’, data currently shared on these repositories are burning fossil fuels. This tension between the social value and environmental cost of sharing can be minimised by sharing only files that are truly needed (Samuel and Lucivero, 2020). This is particularly pertinent for human brain imaging, in which there is often unnecessary duplication of data (pre)processing by individual users. This is not only resource-inefficient, but can also cause problems with reproducibility of results (Botvinik-Nezer et al., 2020). Sharing appropriately documented preprocessed data and derivatives, as opposed to raw data, could help reduce the footprint of sharing, while retaining scientific value.

Moving forward, we hope that resource and energy use related to data management and sharing will become a core consideration in project design and dissemination. It is also essential that we understand more about the precise environmental costs of acquiring new data versus reusing that which has been publicly shared, in order to make more informed judgement calls (Box 4).

**Box 4.** Assessing the carbon footprint of neuroscience.

It remains uncertain what truly sustainable research pipelines look like: We need to more clearly identify the footprint of our research. LEAF provides guidance and accreditation for sustainable practices in wet labs, including calculators to estimate the emission reductions achieved, and the Organization for Human Brain Mapping’s (OHBM) Sustainability and Environment Action Group is working on developing a ‘carbon calculator’ (Mariette et al., 2021) and best practice recommendations on open, sustainable pipelines for human neuroimaging research. By developing such tools as a community, we hope it will become much easier to identify those behaviours that most affect our research footprint (how many emissions are saved by skipping an overseas conference, versus moving data to a server that is powered by renewables?) complementing similar calculations for personal carbon footprints (Wynes and Nicholas, 2017). However, community efforts to develop best practice recommendations will only succeed if neuroscientists actively contribute to groups and task forces (join the OHBM team here).

## Decarbonising academic communities and institutions

Beyond sustainability in our data collection and analyses, we have a powerful role to play as professional scientists more broadly: both in our own behaviour and by changing the governance of our institutions and communities.

### Flying less

A major contribution to academics’ carbon footprint is the habit of frequent, long-distance air travel to meetings and conferences, which contributes substantially to universities’ emissions (Ciers et al., 2019). A case study shows that around 70% of a single 4-year PhD’s carbon emissions come from air travel (Achten et al., 2013), and skipping a single roundtrip trans-Atlantic flight saves more carbon than eating a fully plant-based diet for a year (Wynes and Nicholas, 2017). Taken together, the total travel emissions for one meeting of the Society for Neuroscience may amount to 22,000 metric tonnes CO_2_ (Nathans and Sterling, 2016), as much as the electricity use of almost 4,000 American homes. Unsurprisingly, a small group of (mostly senior) academics take the vast majority of these flights (Arsenault et al., 2019), giving them the most room and responsibility for improvement.

To fly less, we can all attend fewer meetings in person, and meet in conferences and collaborate locally rather than overseas (Nathans and Sterling, 2016). We can choose transportation wisely: trains and carpooling can cover the same distance with a much smaller carbon footprint. If you must fly, choose economy class, avoid layovers, and combine multiple trips to maximise your flight’s scientific gain (Ciers et al., 2019). Crucially, discuss these considerations with your colleagues and students to create a culture of sustainability, for instance by encouraging carbon-conscious travel policies at your institution. Be very wary of carbon offsets: they rarely neutralise all emissions, are difficult or impossible to scale up, and can give the dangerous impression that a small tax is sufficient to mitigate the impact of flying: it is not (Aron et al., 2020). The best way to reduce emissions is to wean ourselves off flying habits, and to keep fossil fuels in the ground.

Hosting virtual meetings eliminates nearly all of their carbon footprint. Although there is some energy cost in hosting and streaming, these are tiny compared to the aviation footprint of a fully in-person meeting. For instance, switching from in-person to online format reduced CO_2_ emissions of a large geophysics conference to around 0.1% (Klöwer et al., 2020). While this insight is far from new (Aron et al., 2020; Nathans and Sterling, 2016; Ponette-González and Byrnes, 2011), it plays out against a conference landscape now irrevocably changed by the Covid-19 pandemic. Over the last year, many existing conferences have gone virtual (e.g. SfN, FENS, OHBM, CCN), and new initiatives such as WorldWideNeuro and NeuroMatch further illustrate the power of virtual scientific exchange (Achakulvisut et al., 2020; van Viegen et al., 2021). Virtual meetings contribute to diversity by strongly reducing or eliminating financial costs, visa and accessibility hurdles, and time away from home (Sarabipour et al., 2021), and many academics are eager to keep some meetings virtual post-Covid (Rissman and Jacobs, 2020). Many innovations in this space are widely useful: for instance, upvoting questions can replace a post-talk sprint to the microphone, ensuring that the most insightful rather than the loudest voices are heard. There is also the promise of immersive virtual reality where conference participants meet ‘in person’ via their avatars and can interact one-on-one or in small groups for discussion or even social events (see, for example, Engage VR). This technology is evolving fast, and the prospect in the not-too-distant future is of lifelike avatars that allow enjoyable as well as scientifically productive social interactions.

Virtual meetings are not a panacea, and many scientists report frustration with all virtual meetings: people experience ‘Zoom fatigue’, disengage and multitask during long days behind the screen, and struggle to balance meeting attendance with ongoing demands at home or in the lab. Many of us crave a return to some in-person social interaction, scientific debate, and collaboration. Academic societies will also need to explore alternative financial models that do not rely on the revenue from wholly in-person annual meetings. Moreover, virtual meetings may pose stronger challenges for early career researchers, who have not yet built strong interpersonal networks – although it is interesting that online-only conferences such as NeuroMatch have been spearheaded by the early career community (Achakulvisut et al., 2020), including the development of guidelines on running online meetings (Achakulvisut et al., 2021).

**Figure 3.**
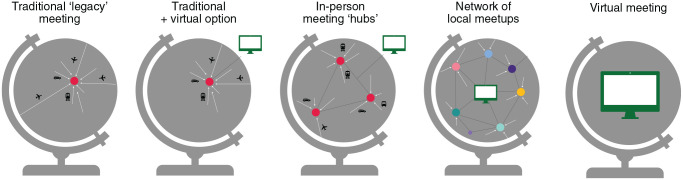
Possible formats for scientific meetings. As scientific meetings prepare for a post-Covid era, we call on conference organisers and attendees to work towards sustainable formats.

Crucially, we can have the best of both worlds: now is the perfect time to rethink how we interact as a community, and integrate virtual components into our post-Covid scientific meetings (Figure 3). One obvious approach is the hybrid meeting with both an in-person and a virtual component, which increases accessibility and reduces long-distance flights. Even more promising is a meeting composed of ‘hubs’ in different locations, strategically placed to minimise travel distance (Klöwer et al., 2020), which strongly reduces carbon emissions without the loss of a large community gathering. Taking these ideas further, we can consider a network of distributed local meetups: an individual scientist or department provides a lecture hall to show streamed talks, books small rooms for one-on-one contact with meeting attendees elsewhere, and hosts social gatherings and meals. Such a distributed model, recently trialled at the NeuroMatch 4.0 conference, allows any location with sufficient interest (and a bit of space) to tune in to large meetings. This combines strong local collaboration and face-to-face interaction with a worldwide virtual community – at a fraction of the emissions and cost. Local meetups have the additional benefit of being resilient and adaptable in the face of changing Covid numbers: when in-person meetings are restricted in one country, this only affects the conference experience of a small number of scientists.

In sum, returning to legacy meetings has strong drawbacks: they cause unsustainable levels of carbon emissions from long-haul flights, limit accessibility to those who can easily travel, and cause jet lag for scientists from different time zones. By tackling the technical and sociological challenges associated with virtual or distributed meetings (in collaboration with professional conference organisers and developers of virtual meeting tools), we can make our scientific community more low-carbon, inclusive, diverse, and run at a fraction of the cost.

### Advocate for sustainability in your institution

Beyond our role in the global neuroscience community, we can use our voice as students and staff members of universities, clinics, and research institutions. If your institution has a sustainability team or office, join them; if not, start one. Faculty governance should demand accurate yearly carbon bookkeeping and concrete plans for emissions reduction across university activities: campus and laboratory operations, food and waste, compute resources and travel, as well as sustainable finance and banking, divestments of endowments and pension schemes from fossil fuels, and energy production. Most of us as we advance in our careers find ourselves starting to engage with the administrative machinery of the institution, and here we have the opportunity to shift focus, influence decisions, and steer resources in a direction that seems important. Although such conversations may not effect immediate change, we can provide a background of constant pressure and concern, normalising action on environmental issues.

In other areas where we have influence, such as on funding panels, we can also speak up about the need for climate-related research and to reduce the footprint of funded projects. UKRI (the UK national taxpayer-funded grant agency) has led in this by developing a comprehensive sustainability policy, including a net-zero target of 2040, and requirements for grant applications to demonstrate that environmental targets have been addressed. It is increasingly likely that journals and funding bodies will ask applicants to address the footprint of their proposed research, though more frameworks and tools are required to facilitate both the quantification and implementation of this. In the meantime, positively and proactively explaining how you have done so may give a competitive edge. If you sit on funding panels, ask whether the sustainability aspects of applications are incorporated into funding decisions – and if not, why not? These considerations may ultimately be integrated in mandatory ‘resource use’ reviews, akin to ethical review boards for human and animal experiments, or explicitly integrated into ethical reviews.

## Changing minds: how scientists can influence society

Up to now we have discussed the local actions we can take to decarbonise our own scientific and academic lives, but as scientists we also have the ability to exert wider influence via our communication channels with educators, the general public, and policymakers. Below, we offer some suggestions for how to do this.

### Research

Many neuroscientists study decision-making and social behaviour. These fields are ideally placed to generate critical new insights into the neural and psychological processes underpinning pro-environmental behaviour and social change. Crucially, knowledge exchange with students, the public, and policymakers should be an ultimate goal to turn scientific insights into societal change. How can we encourage people to engage in behaviours such as reduce meat or flying, and increase walking and cycling (Steg and Vlek, 2009)? How do emotions shape the way we update our beliefs and turn information into actions (Brick et al., 2021)? How can group dynamics facilitate rapid social change (Hauser et al., 2014)? If your lab could answer such questions, consider leveraging your experimental skillset. One can dip toes in the water without fully re-orienting a lab’s purpose: one of us recently collaborated with ecology colleagues to explore the effect of campus biodiversity on student mental health. Going further, some may even take their research in a new direction, as Adam Aron at UCSD has recently done. While it can feel frightening to step out of one’s comfort zone, university faculty are arguably in a unique, secure position that allows pivoting to research with a real-world impact.

### Educators

Many scientists, particularly those based in universities, are involved with education, and many also have connections with schools as educators or parents. There is an appetite among young people for information about climate change, as evidenced by the rapid spread of the school strikes movement started by the teen climate activist Greta Thunberg in 2018 (Boulianne et al., 2020). As universities are increasingly student-centred, communities of carbon-conscious students across departments can demand courses centred on the climate crisis. Schools frequently invite scientists to give talks and may be receptive to offers to talk about climate change. At university level, courses on climate change are springing up. Those neuroscientists in psychology or cognitive science can contribute a psychological dimension (https://www.teachgreenpsych.com/; https://www.apa.org/science/about/publications/climate-change).

Ad hoc lectures are often welcomed by organisers of more general seminar series. It may seem daunting to give talks about a subject one isn’t expert in. However, the climate science required to support a climate talk is rather grimly simple and is readily accessed via the online summaries for policymakers compiled by the Intergovernmental Panel on Climate Change (IPCC) (the latest is available here). Also, general talks about technical matters are sometimes better given by non-specialists, as they convey a clearer overview.

### Communicating with the general public

Scientists can affect the world beyond institutions via their public communication efforts, and as practised communicators we are an important tool in the fight against climate change. But why should neuroscientists, as opposed to climate scientists, engage in public communication of climate change science? One argument is that we are perceived as not just intelligent and informed but also impartial, which may give us added credibility. Like climate scientists, we also understand the dynamics of exponential and cumulative processes, and have data literacy that can help clarify complex scientific information. One suggested way to start the process of giving talks is to contact your local environmental group with an offer. While this may seem like preaching to the converted, if the talk is engaging it is likely to lead to further invitations to other fora. Local leisure clubs and church groups also often welcome suggestions for speakers.

What message should one give, in a public talk? As scientists we expect to provide factual information, but evidence refutes the simplistic ‘information deficit model’, by which climate inaction is entirely explained by an ignorance that we can correct with education (Bauer et al., 2007). The picture is far more nuanced (Zhao and Luo, 2021), as education interacts with people’s pre-existing attitudes and socio-political affiliations (Taube et al., 2021). For example, those who are politically conservative are more likely to be climate sceptics (McCright and Dunlap, 2003), although this may vary by country (Hornsey et al., 2018). Audiences are unlikely to be completely swayed by facts alone, but the facts are nevertheless important to convey, especially as the general public are surprisingly ignorant of the science of climate change (Ranney and Clark, 2016).

The alternative is to influence people’s attitudes via their emotions. Here again, research suggests that the obvious, simplistic approach, in this case to evoke fear, anger, or shame, is potentially counter-productive. Emotions are linked to motivation and action in complex ways, which may also change over time, and with the audience (Chapman et al., 2017). For example, while fear might cause some to take positive action, it may cause others to adopt a denialist attitude (Stern, 2012) and the most effective messaging for a given individual may depend on their pre-existing stance (Hine et al., 2016). Thus, a talk to a general audience should ideally contain a mixture of factual, emotive, and practical information, while also stressing the urgency and importance of collective action, finding a balance between too much fear (leading to denial, despair, or paralysis) and too much hope (leading to complacency). One approach used by the activist group Extinction Rebellion has been to produce a two-part talk, the ‘Heading for Extinction’ talk, in which the first half aims to shock and the second half to galvanise. Obviously the audience demographic needs to be taken into account too, as a different approach is needed with, say, teenagers than with hedge fund managers.

And finally, communicating with the public need not involve getting on a podium in an organised setting. Another important route is via social media, where a lively Facebook or Twitter thread may attract hundreds of readers. It is important to keep social media communications short, friendly, respectful, and factual, remembering that you are writing not for the overt climate deniers in the thread, who will not likely be swayed, but for the silent majority who read and ponder.

### Communicating with politicians and policymakers

Most of us recognise that the climate and ecological crisis is not going to be solved by a population of well-meaning individuals, however large, in the absence of definitive top-down action from world leaders. Leaders, in turn, recognise that they cannot take action without the support of the populace. A two-pronged approach is thus needed: one to influence individuals, as described above, and the other to influence politicians and other societal movers and shakers.

One route for scientists to influence politicians is to contact a local political representative, which in democracies is open to all citizens. Elected representatives are sensitive to the opinions of their constituents, but as one UK member of parliament remarked recently to one of us, ‘my inbox isn’t full of people complaining about the climate crisis, they’re complaining about potholes’. Communications to politicians are therefore more likely to be effective if they are numerous, and this is one practical action that can be suggested at a public talk. Interestingly, recent public polling data in the United Kingdom suggests the tide is turning on how many voters see environmental issues as their top priority, with record numbers ranking sustainability as more important over the economy. However, voters need to let their elected representatives know how strongly they feel.

Scientists may also encounter politicians in other arenas in the course of their academic work, for example when they present to select committees. Although the subject at hand may be something else entirely, the opportunity to engage directly with politicians can be exploited to express concern about the climate and ecological emergency and the slow pace of progress. Senior scientists in particular can have great influence in the public sphere because of their reputations. For example, the renowned climate scientist Chris Rapley recently resigned his position on the Science Museum Advisory Board, citing his disagreement with the museum’s ongoing policy of accepting sponsorship from oil and gas companies. Such acts can provide a highly visible statement that puts pressure on wielders of power to change their practices.

### Communicating with colleagues and friends

Most of our interactions with other people take place outside of the formal frameworks described above. Here, we also have the opportunity to have an influence, this time via social affiliation and the tendency of people to align their views with those of their in-group.

As scientists, most of our interactions are with colleagues and students, who can be gently and repeatedly reminded of the reality of climate breakdown, while recognising that haranguing doesn’t change minds. One way to achieve this is not to cajole or persuade but simply to lead by example, making lifestyle changes that are visible to others, and hoping these spread by ‘social contagion’. Another is to frequently express personal concern about the climate emergency, for example in talks, and highlight particular actions you yourself have taken. Interview candidates can be asked about their sustainability plans with respect to their own research groups or departments. The more the issue is talked about, and such discussion is normalised, the sooner we can move from ‘is it happening?’ to ‘what should we do?’. As the renowned climate scientist and communicator Katherine Hayhoe has noted in her TED talk, ‘The most important thing you can do to fight climate change: *talk about it*’.

## Conclusion

The climate crisis and ecological emergency have never been more urgent. With each day that passes, the carbon in our atmosphere goes relentlessly up, and biodiversity crashes relentlessly down. Neuroscientists, just like everybody else, contribute to these problems, from the environmental costs of what we do in the lab to how we attend conferences. But as professional scientists, we are well placed to systematically and precisely measure the footprint of our research activities and make evidence-based decisions on what needs to change in our research practices.

We call on all neuroscientists, not just those interested in sustainability, to make these changes a matter of the most urgent priority. If we do not lead from the front, how can we expect members of the public at large to support the far-reaching societal shifts that are needed to address the climate crisis? We also call on neuroscientists to become ambassadors for climate action, in their institutions, and in wider society. Many of us hold the reins of power on university committees, and funding panels. From campaigns to decarbonise your institution’s energy supply, to campaigns for meat-free campuses, there are many ways in which a combination of top-down commitment from senior academics, and bottom-up demand from students and staff, can change your institution for the better. It is also critical that those of us who have benefitted the most from the historical, carbon intensive system are also those that carry the lion’s share of the burden of the transition to more sustainable practices.

Most important of all: *talk about it*. Discuss the sustainability implications of research practices within your lab, your department, in meetings, and at conferences. Tell your colleagues how worried you are. Speak to your political representatives. Confronting the ‘inconvenient truth’ of the biggest challenge humanity has ever faced is frightening. We have found that talking about the climate crisis, with each other, with colleagues – in fact, with pretty much anyone – helps us feel less isolated. Speaking out about the climate crisis can also create hope, in finding others who also want to act (Box 5).

**Box 5.** Recommended reading.

• ‘The Garden Jungle’ by Dave Goulson, recommended by C.L.R. Authored by my Sussex colleague and bee expert Prof Dave Goulson, an enchanting journey through the insect life observed in his own back garden, and the damage we are wreaking on invertebrates, upon whom we depend for food and healthy ecosystems. • ‘The Ministry for the Future’ by Kim Stanley Robinson, recommended by A.E.U. A captivating work of climate fiction (‘cli-fi’). It beautifully describes a detailed and well-researched set of potential solutions that may inspire concrete global change. • ‘The Uninhabitable Earth’ by David Wallace Wells, recommended by K.J.J. This is hard-hitting and somewhat catastrophic but very galvanising. • ‘Saving Us’ by Katherine Hayhoe, recommended by A.E.U. A top climate communicator lays out effective strategies for bridging political divides and engaging in meaningful, hopeful climate conversations. • Take a walk in a setting with nature, recommended by M.F. This isn’t a book, but with the volume of climate crisis materials in the news, it’s good to take a break from the news and reading, and indulge in the natural settings we’re fighting to protect.

And there *is* still hope that we can avert the worst possible outcomes. But the window for action is very rapidly closing, and so – neuroscientists – we must act on the climate crisis and ecological emergency. Fast.

## References

[bibr1-23982128221075430] Achakulvisut T Ruangrong T Bilgin I , et al. (2020) Improving on legacy conferences by moving online. eLife 9(1): e57892. 32308195 10.7554/eLife.57892 PMC7170649

[bibr2-23982128221075430] Achakulvisut T Ruangrong T Mineault P , et al. (2021) Towards democratizing and automating online conferences: Lessons from the neuromatch conferences. Trends in Cognitive Sciences 25(4): 265–268. 33608214 10.1016/j.tics.2021.01.007

[bibr3-23982128221075430] Achten WMJ Almeida J Muys B (2013) Carbon footprint of science: More than flying. Ecological Indicators 34(1): 352–355.

[bibr4-23982128221075430] Alves J Sargison FA Stawarz H , et al. (2020) A case report: Insights into reducing plastic waste in a microbiology laboratory. Access Microbiology 3(3): 000173. 34151149 10.1099/acmi.0.000173 PMC8209715

[bibr5-23982128221075430] Anthony LFW Kanding B Selvan R (2020) Carbontracker: Tracking and predicting the carbon footprint of training deep learning models. arXiv 2007.03051. Available at: https://arxiv.org/abs/2007.03051

[bibr6-23982128221075430] Aron AR Ivry RB Jeffery KJ , et al. (2020) How can neuroscientists respond to the climate emergency? Neuron 106(1): 17–20. 32272064 10.1016/j.neuron.2020.02.019

[bibr7-23982128221075430] Arsenault J Talbot J Boustani L , et al. (2019) The environmental footprint of academic and student mobility in a large research-oriented university. Environmental Research Letters 14(1): 095001.

[bibr8-23982128221075430] Balmford A Fisher B Mace GM , et al. (2020) Analogies and lessons from COVID-19 for tackling the extinction and climate crises. Current Biology 30(17): R969–R971. 32898490 10.1016/j.cub.2020.06.084 PMC7321052

[bibr9-23982128221075430] Bauer MW Allum N Miller S (2007) What can we learn from 25 years of PUS survey research? Liberating and expanding the agenda. Public Understanding of Science 16(1): 79–95.

[bibr10-23982128221075430] Boto E Holmes N Leggett J , et al. (2018) Moving magnetoencephalography towards real-world applications with a wearable system. Nature 555(1): 657–661. 29562238 10.1038/nature26147 PMC6063354

[bibr11-23982128221075430] Botvinik-Nezer R Holzmeister F Camerer CF , et al. (2020) Variability in the analysis of a single neuroimaging dataset by many teams. Nature 582(1): 84–88. 32483374 10.1038/s41586-020-2314-9 PMC7771346

[bibr12-23982128221075430] Boulianne S Lalancette M Ilkiw D (2020) ‘School Strike 4 Climate’: Social media and the international youth protest on climate change. Media Communication 8(2): 208–218.

[bibr13-23982128221075430] Brick C Bosshard A Whitmarsh L (2021) Motivation and climate change: A review. Current Opinion in Psychology 42(1): 82–88. 33992934 10.1016/j.copsyc.2021.04.001

[bibr14-23982128221075430] Cascini A Bortolini M Botti L , et al. (2013) Life cycle assessment of a commercial refrigeration system under different use configurations. In: Proceedings of the 18th Summer School ‘Francesco Turco’ 2, Senigallia, pp. 352–357. Available at: http://summerschool-aidi.it/edition-2015/images/ancona2013/articoli/non_presentati/articolo16_np.pdf

[bibr15-23982128221075430] Chapman DA Lickel B Markowitz EM (2017) Reassessing emotion in climate change communication. Nature Climate Change 7(1): 850–852.

[bibr16-23982128221075430] Ciers J Mandic A Toth LD , et al. (2019) Carbon footprint of academic air travel: A case study in Switzerland. Sustainability 11(1): 80.

[bibr17-23982128221075430] Dockx P (2015) Lab ventilation and energy consumption. In: Dittrich E (ed.) The Sustainable Laboratory Handbook. Chichester: John Wiley & Sons Ltd, pp. 363–378.

[bibr18-23982128221075430] Frith U (2020) Fast lane to slow science. Trends in Cognitive Sciences 24(1): 1–2. 31744772 10.1016/j.tics.2019.10.007

[bibr19-23982128221075430] Fuss S Canadell JG Peters GP , et al. (2014) Betting on negative emissions. Nature Climate Change 4(1): 850–853.

[bibr20-23982128221075430] Glasser MF Smith SM Marcus DS , et al. (2016) The Human Connectome Project’s neuroimaging approach. Nature Neuroscience 19(1): 1175–1187. 27571196 10.1038/nn.4361 PMC6172654

[bibr21-23982128221075430] Goyal-Kamal Feld B Schmidt V , et al. (2021) CodeCarbon: Estimate and track carbon emissions from machine learning computing. Zenodo. Available at: 10.5281/zenodo.4699491

[bibr22-23982128221075430] Hafer M (2017) Quantity and electricity consumption of plug load equipment on a university campus. Energy Efficiency 10(1): 1013–1039.

[bibr23-23982128221075430] Hallegatte S Green C Nicholls RJ , et al. (2013) Future flood losses in major coastal cities. Nature Climate Change 3(1): 802–806.

[bibr24-23982128221075430] Haugen RK (2020) Laboratory Hood Energy Savings: The Low-Hanging Fruit. ACS Chemical Health & Safety 27(2): 125–128.

[bibr25-23982128221075430] Hauser OP Rand DG Peysakhovich A , et al. (2014) Cooperating with the future. Nature 511(1): 220–223. 25008530 10.1038/nature13530

[bibr26-23982128221075430] Hine DW Phillips WJ Cooksey R , et al. (2016) Preaching to different choirs: How to motivate dismissive, uncommitted, and alarmed audiences to adapt to climate change? Global Environmental Change 36(1): 1–11.

[bibr27-23982128221075430] Hornsey MJ Harris EA Fielding KS (2018) Relationships among conspiratorial beliefs, conservatism and climate scepticism across nations. Nature Climate Change 8(1): 614–620.

[bibr28-23982128221075430] Intergovernmental Science-Policy Platform on Biodiversity and Ecosystem Services (2019) Summary for policymakers of the global assessment report on biodiversity and ecosystem services. Zenodo. Available at: 10.5281/zenodo.3553579

[bibr29-23982128221075430] Klöwer M Hopkins D Allen M , et al. (2020) An analysis of ways to decarbonize conference travel after COVID-19. Nature 583(1): 356–359. 10.1038/d41586-020-02057-2 32669689

[bibr30-23982128221075430] Littlejohns TJ Holliday J Gibson LM , et al. (2020) The UK Biobank imaging enhancement of 100,000 participants: rationale, data collection, management and future directions. Nature Communication 11(1): 2624. 10.1038/s41467-020-15948-9 PMC7250878 32457287

[bibr31-23982128221075430] McCright AM Dunlap RE (2003) Defeating Kyoto: The Conservative Movement’s Impact on US. Climate Change Policy. Social Problems 50(3): 348–373.

[bibr32-23982128221075430] Mariette J Blanchard O Berné O , et al. (2021) An open-source tool to assess the carbon footprint of research. arXiv 2101.10124. Available at: https://arxiv.org/abs/2101.10124

[bibr33-23982128221075430] Marković D Mizrahi A Querlioz D , et al. (2020) Physics for neuromorphic computing. Nature Reviews Physics 2(1): 499–510.

[bibr34-23982128221075430] Masson-Delmotte V Zhai P Pörtner HO , et al. (2018) IPCC, 2018: Summary for policymakers. In: Global Warming of 1.5°C. An IPCC Special Report on the Impacts of Global Warming of 1.5°C above Pre-Industrial Levels and Related Global Greenhouse Gas Emission Pathways, in the Context of Strengthening the Global Response to the Threat of Climate Change Sustainable Development, and Efforts to Eradicate Poverty. Geneva: World Meteorological Organization, p. 32.

[bibr35-23982128221075430] Mbow C Rosenzweig C Barioni LG , et al. (2019) Food security. In: Shukla PR Skea E Calvo Buendia E , et al. (eds) Climate Change Land. pp. 437–550. Available at: https://www.ipcc.ch/site/assets/uploads/2019/11/08_Chapter-5.pdf

[bibr36-23982128221075430] Nathans J Sterling P (2016) How scientists can reduce their carbon footprint. eLife 5(1): e15928. 27029962 10.7554/eLife.15928 PMC4829415

[bibr37-23982128221075430] Nielsen KS Nicholas KA Creutzig F , et al. (2021) The role of high-socioeconomic-status people in locking in or rapidly reducing energy-driven greenhouse gas emissions. Nature Energy 6(1): 1011–1016.

[bibr38-23982128221075430] Nolan CJ Field CB Mach KJ (2021) Constraints and enablers for increasing carbon storage in the terrestrial biosphere. Nature Reviews Earth & Environment 2(1): 436–446.

[bibr39-23982128221075430] Otto IM Donges JF Cremades R , et al. (2020) Social tipping dynamics for stabilizing Earth’s climate by 2050. Proceedings of the National Academy of Sciences of the United States of America 117(5): 2354–2365. 31964839 10.1073/pnas.1900577117 PMC7007533

[bibr40-23982128221075430] Papadakis M Meiwandi A Grzybowski A (2019) The WHO safer surgery checklist time out procedure revisited: Strategies to optimise compliance and safety. International Journal of Surgery 69(1): 19–22. 31310820 10.1016/j.ijsu.2019.07.006

[bibr41-23982128221075430] Ponette-González AG Byrnes JE (2011) Sustainable science? Reducing the carbon impact of scientific mega-meetings. Ethnobiology Letters 2(1): 65–71.

[bibr42-23982128221075430] Ranney MA Clark D (2016) Climate change conceptual change: Scientific information can transform attitudes. Topics in Cognitive Science 8(1): 49–75. 26804198 10.1111/tops.12187

[bibr43-23982128221075430] Rillig MC Lehmann A Bank MS , et al. (2021) Scientists need to better communicate the links between pandemics and global environmental change. Nature Ecology & Evolution 5(1): 1466–1467. 34475572 10.1038/s41559-021-01552-7

[bibr44-23982128221075430] Rissman L Jacobs C (2020) Responding to the climate crisis: The importance of virtual conferencing post-pandemic. Collabra: Psychology 6(1): 17966.

[bibr45-23982128221075430] Rizan C Bhutta MF Reed M , et al. (2021) The carbon footprint of waste streams in a UK hospital. Journal of Cleaner Production 286(1): 125446.

[bibr46-23982128221075430] Rolnick D Donti PL Kaack LH , et al. (2019) Tackling climate change with machine learning. arXiv 1906.05433. Available at: https://arxiv.org/abs/1906.05433

[bibr47-23982128221075430] Samuel G Lucivero F (2020) Responsible open science: Moving towards an ethics of environmental sustainability. Publications 8(4): 54. 10.3390/publications8040054 PMC7616685 39399694

[bibr48-23982128221075430] Sarabipour S Khan A Seah YFS , et al. (2021) Changing scientific meetings for the better. Nature Human Behaviour 5(1): 296–300. 10.1038/s41562-021-01067-y 33723404

[bibr49-23982128221075430] Schwartz R Dodge J Smith NA , et al. (2020) Green AI. Communication of the ACM 63(12): 54–63.

[bibr50-23982128221075430] Steg L Vlek C (2009) Encouraging pro-environmental behaviour: An integrative review and research agenda. Journal of Environmental Psychology 29(3): 309–317.

[bibr51-23982128221075430] Stern PC (2012) Fear and hope in climate messages. Nature Climate Change 2(1): 572–573.

[bibr52-23982128221075430] Takeda T Okamoto M Miyazaki T , et al. (2011) Helium circulation system (HCS) for the MEG, Magnetoencephalography. IntechOpen. Available at: 10.5772/28904

[bibr53-23982128221075430] Taube O Ranney MA Henn L , et al. (2021) Increasing people’s acceptance of anthropogenic climate change with scientific facts: Is mechanistic information more effective for environmentalists? Journal of Environmental Psychology 73(1): 101549.

[bibr54-23982128221075430] The International Brain Laboratory, Bonacchi N Chapuis G , et al. (2019) Data architecture and visualization for a large-scale neuroscience collaboration. bioRxiv 827873. Available at: 10.1101/827873

[bibr55-23982128221075430] van Viegen T Akrami A Bonnen K , et al. (2021) Neuromatch Academy: Teaching computational neuroscience with global accessibility. Trends in Cognitive Sciences 25(7): 535–538. 33994097 10.1016/j.tics.2021.03.018

[bibr56-23982128221075430] Wynes S Nicholas KA (2017) The climate mitigation gap: Education and government recommendations miss the most effective individual actions. Environmental Research Letters 12(1): 074024.

[bibr57-23982128221075430] Zak JD Wallace J Murthy VN (2020) How neuroscience labs can limit their environmental impact. Nature Reviews Neuroscience 21(1): 347–348. 10.1038/s41583-020-0311-5 PMC13020460 32385420

[bibr58-23982128221075430] Zhao J Luo Y (2021) A framework to address cognitive biases of climate change. Neuron 109(22): 3548–3551. 34555315 10.1016/j.neuron.2021.08.029

